# Phage vB_Ec_ZCEC14 to treat antibiotic-resistant *Escherichia coli* isolated from urinary tract infections

**DOI:** 10.1186/s12985-024-02306-0

**Published:** 2024-02-16

**Authors:** Nedaa M. Ismael, Mohamed Azzam, Mohamed Abdelmoteleb, Ayman El-Shibiny

**Affiliations:** 1 Center for Microbiology and Phage Therapy, Zewail City of Science and Technology, 12578 Giza, Egypt; 2 Department of Botany, Faculty of Science, Mansoura University, 35516 Mansoura, Egypt; 3 Faculty of Environmental Agricultural Sciences, Arish University, 45511 Arish, Egypt

**Keywords:** *Escherichia coli*, Bacteriophage, Phage therapy, Urinary tract infections, Multi-drug resistance (MDR)

## Abstract

*Escherichia coli* is a commensal bacterial species in the human gastrointestinal tract; however, it could be pathogenic and cause severe infections in intra and extra-intestinal sites. Uropathogenic *E. coli* accounts for 80–90% of urinary tract infections that can result in urosepsis and septic shock. Consequently, multidrug-resistant uropathogenic *E. coli* poses a considerable risk to the healthcare system worldwide. Phage therapy is demonstrated as an optimistic solution to over-the-counter antibiotics that contribute to the global issue of multidrug-resistant bacteria. This study aims to isolate a novel phage that could be implemented to cure urinary tract infections mediated by multidrug-resistant *E. coli*. Twenty-seven *E. coli* isolates were collected from patients with urinary tract infections to assess the antibacterial efficacy of phage vB_Ec_ZCEC14. Phage kinetics were encountered against the *E. coli* strain (EC/4), in addition to evaluating phage stability under various temperatures, pH values, and UV exposure periods. Full genome sequencing and morphological analysis were conducted for further phage characterization, which revealed that phage vB_Ec_ZCEC14 belongs to the family *Straboviridae*. Phage vB_Ec_ZCEC14 showed thermal tolerance at 80 ℃, pH stability between pH 3 and pH 12, and endurance to UV exposure for 45 min. The phage-host interaction results revealed that phage vB_Ec_ZCEC14 has strong and steady antibacterial action at lower concentrations (MOI 0.1). The study findings strongly indicate that phage vB_Ec_ZCEC14 holds significant promise as a potential therapeutic alternative for treatment of antibiotic-resistant uropathogenic *E. coli.*

**Supplementary Information:**

The online version contains supplementary material available at 10.1186/s12985-024-02306-0.

## Introduction

Urinary tract infections (UTIs) are quite common these days due to many bacterial infections. Urinary tract infections (UTIs) are a major cause of disease worldwide, accounting for 404.6 million cases in both males and females each year and 236,786 fatalities [1]. It accounts for 13–19% of cases and poses a considerable risk of developing urosepsis, which occurs in up to 25% of patients [2]. Some urinary tract infections damage the urethra and bladder. Upper urinary tract infections can be fatal if bacteria from the infected kidneys enter the bloodstream, a condition known as urosepsis [3]. Urinary tract stones could be peripheral to UTIs, as uropathogens can promote stone origination. Patients with urolithiasis, who are suffering from UTIs, are more likely to encounter severe complications following surgical operations, such as systemic inflammation, postoperative infection, and death due to septic shock [4, 5].

While Gram-negative bacilli cause most of the urosepsis incidents, *E. coli* accounts for 50% of the cases, followed by *Enterobacter* spp. and *Klebsiella* spp. (15%), *Proteus* spp. (15%), and *Pseudomonas aeruginosa* (5%). Whereas Gram-positive bacteria only comprise about 15% of the cases [6]. *E. coli* is predominant in the large intestine of warm-blooded mammals and results in widespread bacterial infections in humans and livestock [7]. *E. coli* strains may be commensal, residing in a symbiotic relationship that confers resistance to harmful species, or they could be pathogenic and cause severe infections of intestinal and extraintestinal locations [8]. *E. coli* is responsible for up to 90% of UTI incidences and is often detected in the gastrointestinal microbiota of the same host [9, 10].

Extraintestinal pathogenic *E. coli* (ExPEC) bacteria are categorized into uropathogenic *E. coli* (UPEC), neonatal meningitis *E. coli*, and septicemic *E. coli* [11]. *E. coli* causes UTIs both in the general public and in hospitals, particularly originated by biofilm-producing *E. coli* and extended-spectrum beta-lactamase *E. coli* (ESBL) [12, 13]. UPEC is the most prevalent *E. coli* type of ExPEC among UTIs patients [14]. Oral antibiotics e.g. fluoroquinolones, rimethoprim-sulfamethoxazole and cephalosporins have been traditionally used to treat UTIs resulted by *E. coli* [15]. Unfortunately, these drugs’ effectiveness has decreased recently due to widespread usage and the emergence of resistance [16].

Antibiotic-resistant bacteria have become an alarming public health concern in the last few decades [17]. Finding alternatives to treat bacterial infections has become a key research priority because of the increasing prevalence of Multi-Drug-Resistant (MDR) bacterial infections, particularly in hospitals. In addition to standard antibiotics, innovative treatment techniques must be established quickly [18, 19] The imminent deterioration of current antibiotics has rekindled a global interest in phage therapy [20]. Alternatively, phage therapy is considered as a potential therapeutic solution that may reduce the growing antibiotic resistance, either as a substitute for antibiotics or in conjunction with antibiotic treatment [21]. Bacteriophage therapy was reported to be a safe, competent, and simple substitute to deal with the outgrowing issues accompanying MDR bacteriaIn clinical research conducted in the late 1950s, 607 individuals who had not responded to conventional antibiotic therapy were treated with bacteriophages, and no adverse consequences were noted [21, 22]. Prof. Stefan Slopek, who had a pioneering role in bacteriophage research in Poland, claimed that adverse outcomes due to phage therapy were rarely seen among 550 cases screened at the Hirszfeld Institute between 1981 and 1986. In addition, more than one thousand patients were treated with bacteriophage between 1954 and 1987, and their cases reported a success rate of 84–97% [23, 24].

. Phages lyse their host bacterial cells without affecting the non-host species. Following the elimination of the bacterial host, the count of the phage population reduces as well, maintaining the microbiota’s stability and variety [25].

In this study, we isolated a new phage vB_Ec_ZCEC14, to inhibit the multidrug-resistant UPEC *EC*/04. The results of our study showed vB_Ec_ZCEC14 ability to lyse MDR *E. coli* isolated from UTIs. This is a step towards identifying an alternative therapy for multi-drug resistant UPECs.

## Materials & methods

### Bacterial isolation

Thirty-two UTIs *E. coli* isolates were collected from different human urine samples at Suez Canal University Hospital and gifted to Center for Microbiology and Phage Therapy at Zewail City of Science and Technology. In addition, ten *E. coli* strains from *E. coli* reference (ECOR) collection were used in this study. Bacterial isolates were identified and confirmed through culturing on differential selective Eosin Methylene Blue media (EMB-Oxoid, UK) and MacConkey agar (Oxoid, UK) prior to additional subcultures. Furthermore, the bacterial identity was validated and confirmed by PCR, in which QIAamp DNA Mini kit (Qiagen, Germany) was used for DNA extraction from bacterial isolates. Furthermore, size of the PCR product was determined by running it on an agarose gel (1.0% (w/v) agarose). Tryptone Soy Agar (TSB-Merck, Germany) was used in all study experiments. Tryptone Soy Broth (Merck, Germany) supplemented with 20–25% (w/v) glycerol was used to preserve all isolates at -80 °C until they were needed.

### 16 S ribosomal RNA gene sequencing

*E. coli* isolate (*EC*/4) was identified through PCR amplification using the primers F (5′- AAGAAGCACCGGCTAACTCC − 3′) - R (5′- CGCTTCTCTTTGTATGCGCC − 3′) and 16 S rRNA gene sequencing using 27 F and 1492R primers. 16 S rRNA sequencing was conducted using the automated fluorescent-DNA sequencer (Applied Biosystem, model 373/USA). Finch TV (digitalworldbiology.com/FinchTV/; date of access: 11-AUG-2022) was used to check quality and clean the sequence. BLASTn (Altschul et al., 1990) was used to check the homology in the bacterial 16 S rRNA database (Access date: Aug 11, 2022). The sequenced 16 S rRNA gene of *EC*/4 has been deposited into the NCBI GenBank database (Accession Number OP205141).

### Detection of resistance and virulence genes by PCR

Antibiotic resistance and virulence genes were screened in the tested *E. coli* isolates. They were cultured for 4 h in Tryptone Soy Broth (TSB-Merck, Germany). PCR amplification was then conducted to detect the following virulence genes: fimH, traT, blaTEM, blaSHV, blaCTX, and tetA. All the primer sequences, annealing temperatures, and amplicon sizes are provided in Table 1.

**Table 1:** Conditions and primers for PCR amplification of species, antibiotic resistance genes, and virulence markers in *E. coli* isolates

Virulence factor	Target gene	Primer	Sequence (5` to 3`)	Length (bp)	Annealing temperature (°C)	Amplicon Size (pb)	References
traT	traT	traT-F traT-R	GGTGTGGTGCGATGAGCACAG CACGGTTCAGCCATCCCTGAG	21 21	60	290	[26]
fimH	fimH	fimH-F fimH-R	CATTCGCCTGTAAAACCGCC ATAACACGCCGCCATAAGCC	20 20	60	207	[27]
blaCTX	blaCTX	blaCTX-F blaCTX-R	TTTGCGATGTGCAGTACCAGTAA CGATATCGTTGGTGGTGCCATA	23 23	55	544	[28]
tetA	tetA	tetA-F tetA-R	TTGGCATTCTGCATTCACTC GTATAGCTTGCCGGAAGTCG	20 20	60	494	[29]
blaTEM	blaTEM	TEM-F TEM-R	ATAAAATTCTTGAAGACGAAA GACAGTTACCAATGCTTAATCA	19 19	50	1150	[30]
blaSHV	blaSHV	SHV-F SHV-R	CACTCAAGGATGTATTGTG TTAGCGTTGCCAGTGCTCG	19 19	50	885	[31]

### Antibiotic susceptibility assay

Antibiotic susceptibility of *E. coli* isolates was tested following National Committee for Clinical Laboratory Standards (NCCLS) guidelines using disc diffusion method [32]. Among the 32 urine isolates, only 25 of them were confirmed as *E. coli* and they were subjected to a panel of antibiotic discs including: Amoxicillin (AMC; 30 μg), Ceftriaxone (CRO; 30 μg), Chloramphenicol (C; 30 μg), Ciprofloxacin (CIP; 5 μg), Doxycycline (DO; 30 μg), Etrapenem (ETP; 10 μg), Gentamicin (CN; 10 μg), Nalidixic acid (NA; 30 μg), Nitrofurantoin (F; 300 μg), Norfloxacin (NOR; 10 μg), and Tetracycline (TE; 10 μg). Then, the diameter of inhibition zone for each antibiotic was measured and interpreted [33, 34] in reference to the Clinical and Laboratory Standards Institute [35]. Accordingly, *E. coli* isolates were classified into resistant, intermediate resistant, or susceptible.

### Bacteriophage isolation and amplification

Twenty sewage-water samples were collected from Giza area, Egypt to isolate bacteriophages. Sewage water samples were pre-incubated overnight with a cocktail of confirmed *E. coli* isolates to enrich the phage population [36]. The samples were centrifuged at 4 ℃ for 15 min at 5000 rpm [37, 38] and chloroform (1%) was added to lyse bacterial cells having intracellular phages. To examine the lysis efficacy of the isolated phages to bacterial hosts, spot assay was conducted on a fresh lawn of bacterial isolates on a soft-overlay agar [39, 40]. Seven clear-plaquing phages against different bacterial hosts were subjected to further investigations using the primary bacterial host *EC*/4. Phage purification was performed through four to five isolations of each single plaque. All phage amplifications were conducted in liquid culture of tryptic soya, followed by cooling centrifugation at 5000 rpm for 15 min and the supernatant lysate was collected [41, 42]. Next, the phage count was enumerated by performing a 10-fold serial dilution and spotting 10 μL volume of each dilution in triplicate on a fresh lawn of the bacterial host [43]. The purified phages were stored in SM buffer (pH 7.5) [44]. Phage vB_Ec_ZCEC14 was selected among all the isolated phages according to its broad host range.

### Phage host range analysis

Host range analysis of phage vB_Ec_ZCEC14 was performed using the twenty-five confirmed *E. coli* isolates and ten isolates from ECOR collection. A spot assay was conducted by adding 100 μL volume of a mid-log growing bacterial culture to 4 mL volume of soft agar (0.3% w/v), followed by pouring the mixture onto the top of TSA agar base [45]. Finally, the phage lysate was spotted with volume 10 μL in triplicates on the dried bacterial lawns that were overnight incubated at 37 °C [46].

### Efficiency of plating (EOP)

Efficacy of phage vB_Ec_ZCEC14 against susceptible *E. coli* host strains was carried out as a further assessment. This experiment is conducted using log-phase bacteria under the same conditions of the spotting assay. After an overnight incubation period, the PFUs formed by the phage on each bacterial host were counted and the average was calculated and then divided by the highest observed PFU count to get the EOP [47, 48].

### Frequency of bacteriophage insensitive mutants (BIMs)

To determine the BIMs, the susceptible bacterial host *EC*/4 was treated with phage vB_Ec_ZCEC14 at multiplicity of infection (MOI) concentration 100. The phage suspension was serially diluted and spotted after 10 min of incubation at 37 °C as described before, followed by an overnight incubation. The BIM is calculated as the ratio of viable bacterial counts that resist phage infection to the primary viable bacterial counts [49].

### Pulsed field gel electrophoresis (PFGE)

DNA of phage vB_Ec_ZCEC14 ($$ {10}^{10}$$ PFU/mL) was prepared for PFGE to determine the genome size [50, 51]. In brief, phage capsids and protein were subjected to digestion overnight at 55 °C in lysis buffer (1 mg/mL proteinase K; 100 mM EDTA; 0.2% w/v SDS; 1% w/v N-lauryl sarcosine) with gentle shaking. Agarose-containing DNA slices were washed in washing buffer and placed into wells of a 1% w/v agarose gel. Then, electrophoresis was performed using running buffer (0.5 Tris-borate-EDTA) in a Bio-Rad CHEF DRII system at 200 V (6 V/cm) at 14 °C for 18 h with 30 to 60 s switch time. Lambda DNA markers (Sigma Aldrich, Gillingham, UK) were used to determine the genome size of phage vB_Ec_ZCEC14.

### Bacteriophage morphology

The morphological structure of phage vB_Ec_ZCEC14 was examined using Transmission Electron Microscopy (TEM) at the Medical Research Institute (Alexandria, Egypt). A high concentration of purified phage vB_Ec_ZCEC14 lysate (10^10^ PFU/ml) was loaded into copper grids coated with a Formvar carbon before treatment of grids with 2.5% glutaraldehyde, followed by rinsing and staining with 2% phosphotungstic acid at pH 7.0. After drying, copper grids were examined by TEM (JEOL 1230) to obtain micrographs of phage vB_Ec_ZCEC14 [52].

### In vitro characterization of phage vB_Ec_ZCEC14

Lytic activity of vB_Ec_ZCEC14 against bacterial host strain *EC*/4 was in vitro examined at MOI values: 0.1, 1, and 10 PFU/CFU. It was evaluated against log-phase growing *E. coli EC*/4 (10^8^ CFU/mL) at 37 °C over ten time intervals (0, 10, 20, 30, 40, 60, 75, 90, 120, and 180 min) [53]. In summary, two flasks were prepared, one as a control containing bacterial culture without phage and the other containing the same culture supplemented with bacteriophage at the same MOI. Simultaneously, titers of bacterial control (B), bacterial survival (BS), and plaque-forming units (PFU) were calculated at all time points. Samples of B and BS were subjected to concentration calculations following Miles and Misra method [54]. Briefly, serial dilutions were prepared after mixing 100 μl of the sample with 900 μl TSB. The contents were gently shaken to achieve a consistent distribution of cells and get the required dilution (10^− n^). A 10 μl of each dilution is spotted on a bacterial lawn of the host strain. The agar plates were incubated for 18 to 24 h at 35 to 37 °C. Meanwhile, double-agar overlay plaque assay was implemented to determine PFU titer as described before.

### Bacteriophage pH, temperature, and UV stability

The thermal stability of vB_Ec_ZCEC14 (at a concentration of $$ {10}^{9}$$ PFU/ml) was measured over a period of one hour, using a water bath. The tested temperatures included 4 °C, 20 °C, 37 °C, 50 °C, 60 °C, 70 °C, 75 °C, and 80 °C [55]. Following the standard double layer technique, serial dilutions of phage vB_Ec_ZCEC14 were in triplicate applied on bacterial lawns of host strain EC/4 immediately after incubation, in order to ascertain the phage’s titers [56]. The assessment of the phage’s UV stability was conducted at discrete time points of 15, 30, 45, and 60 min under the UV lamb of the biological safety cabinet with intensity around 253.7 nm. The standard double-layer technique was used to enumerate phage titers. Stability of pH was determined through incubation for 24 h at different pH values (1, 2, 4, 5, 7, 9, 10, 12, and 13) [57].

### DNA sequencing and bioinformatic analysis for phage vB_Ec_ZCEC14

The genomic DNA was extracted using the following platform. First, vB_Ec_ZCEC14 (≈ 10¹° PFU/mL) lysates were incubated with proteinase K solution (100 g/mL dissolved in 10 mM EDTA (pH = 8)). Afterwards, the genomic DNA was extracted using DNA Wizard Kit (Promega, United Kingdom). Illumina MiSeq sequencing was conducted following Nextera tagmentation (Illumina, Cambridge, UK) workflow, generating 150 bp paired end reads. FASTQC [58] was used to check sequencing quality and poor-quality bases were cleaned using PRINSEQ [59]. SPAdes [60] assembler was integrated for de novo assembly of the cleaned data using different K-mers, generating one unique contigs of 165,622 bp length. Quality of de novo assembly was statistically measured using QUAST [61]. ORF finder (Access date: 10 June 2022; available at: www.ncbi.nlm.nih.gov/orffinder/) was implemented to predict open-reading frames. Coding sequences (CDSs) were manually curated through BLASTp [62] of the resulted ORFs against NCBI Protein database (NR). Functional annotation of CDSs was also compared to PATRIC [63] and RASTtk [64] platforms to increase our confidence in the predicted function of coding regions. Genetic maps were generated using SNAPGene highlighting coding genes of assigned functions (GSL Biotech; access date: 11 June 2022; Available at: www.snapgene.com/). The vB_Ec_ZCEC14 genome has been imported into GenBank under the entry number OP168898. PhageLeads explored the presence of any temperate, virulence, and antibiotic resistance genes to check phage suitability for therapeutic applications [65]. In addition, DeepTMHMM was used to detect transmembrane topology among the predicted proteins. Transmembrane proteins play a crucial role in viral pathogenicity and different stages of replication, starting from genome uncoating ending up with viral release [66]. Phylogeny of phage vB_Ec_ZCEC14 was screened using multiple platforms. Highly similar phages to phage vB_Ec_ZCEC14 were characterized by BLASTn [62]. The genome-genome distances were computed using VICTOR phylogeny [67] using the BLASTn top 100 matched phages. Pairwise sequence alignments were performed following Genome-BLAST Distance Phylogeny (GBDP) workflow [68]. In addition, intergenomic pairwise similarities were calculated following VIRDIC workflow [69], in which species (> 95%) and genus (> 70%) thresholds were used as default parameters. Furthermore, Viral Proteomic Tree (ViPTree) [70] was implemented to draw a proteomic tree of vB_Ec_ZCEC14 following tBLASTx (Altschul et al. 1990) genome-wide sequence alignments. Orthologous (signature) genes between vB_Ec_ZCEC14 and closely related phages generated from ViPTree were predicted by CoreGenes 0.5 [71]. Conserved proteins were used to draw a phylogenetic tree in MEGA 11 [72] using top BLASTp matched of other phages. CLUSTAL-W aligner was used to compare the conserved proteins [72] and bootstrap of 1000 replicates and best Maximum Likelihood fit model were the default settings.

## Results

### Bacterial isolation and identification

PCR results validated the identity of *E. coli* isolates using the specific primers (F: 5′- AAGAAGCACCGGCTAACTCC − 3′ and R: 5′- CGCTTCTCTTTGTATGCGCC − 3′) by amplification of a 766-bp band matching a conserved area in 16 S rRNA. *E. coli* strain *EC/*4 was partially sequenced and deposited into GenBank (Acc. No. OP205141). BLASTn results of *EC/*4 sequence showed 98.54% sequence identity to *E. coli* NBRC 102,203 strain.

### Virulence genes

A survey of six pathogenicity genes of *E. coli* (traT, fimH, blaCTX, blaSHV, blaTEM, and tetA) was tested in eight highly MDR isolates using a PCR assay. The results revealed that two isolates have five out of six genes, three isolates have four genes, one isolate has three genes, and the remaining two isolates have two genes.

### Antibiotic susceptibility profile

The sensitivity of bacterial isolates to antibiotics is classified into three categories: susceptible, intermediate resistant, and resistant. Antibiotic susceptibility test of *E. coli* isolates reflected their different sensitivity patterns to the tested antibiotic classes. The results revealed that 8 confirmed *E. coli* isolates are MDR bacteria based on their MAR index as they showed resistance to 5 or 6 classes of antibiotics, as illustrated in Table 2. Three isolates were resistant to Nitrofurantoin (F) and Amoxicillin (AMC); 12 resistant to Tetracycline (TE) and Nalidixic acid (NA); 2 resistant to Chloramphenicol (C); 5 resistant to Ertapenem (ETP); 9 resistant to Norfloxacin (NOR); 10 resistant to Gentamicin (CN); 11 resistant to Doxycycline (DO); and finally 15 resistant to Ceftriaxone (CRO).

**Table 2:** Antibiotic susceptibility of *E. coli* isolates. Abbreviations: MAR: Multi-drug resistant, S: Sensitive, I: Intermediate, R: Resistant, C: Chloramphenicol, CRO: Ceftriaxone, DO: Doxycycline, NOR: Norfloxacin, AMC: Amoxicillin, TE: Tetracycline, CIP: Ciprofloxacin, ETP: Etrapenem, F: Nitrofurantoin, NA: Nalidixic acid and CN: Gentamicin

Antibiotics	C30	CRO30	DO30	NOR10	AMC30	TE10	CIP5	ETP10	F300	NA30	CN10	MAR Index
*E. coli* 1	I	I	I	R	R	S	S	S	I	S	R	**0.2**
*E. coli* 2	S	R	S	R	I	S	R	S	S	R	R	**0.1**
*E. coli* 3	S	R	S	R	I	S	R	S	S	R	S	**0.3**
*E. coli* 4	S	I	R	I	S	R	R	S	S	R	S	**0.3**
*E. coli* 5	I	R	S	R	I	R	R	R	I	R	R	**0.6**
*E. coli* 6	S	I	R	S	S	R	I	S	I	S	S	**0.1**
*E. coli* 7	S	R	R	S	R	R	R	I	R	S	I	**0.5**
*E. coli* 8	S	R	S	S	I	S	S	S	S	S	S	**0.09**
*E. coli* 9	I	R	S	R	I	R	R	I	S	R	R	**0.5**
*E. coli* 10	S	S	I	S	S	S	S	I	R	S	R	**0.1**
*E. coli* 11	I	I	R	I	S	S	S	I	S	S	I	**0.09**
*E. coli* 12	S	R	S	R	S	S	I	I	S	R	I	**0.2**
*E. coli* 13	R	S	R	S	I	R	S	S	S	S	S	**0.2**
*E. coli* 14	S	R	S	R	I	S	R	I	I	R	R	**0.4**
*E. coli* 15	S	R	S	S	S	S	I	I	S	S	I	**0.2**
*E. coli* 16	S	R	R	I	I	R	R	R	I	I	R	**0.5**
*E. coli* 17	S	I	S	S	S	S	S	S	S	R	I	**0.09**
*E. coli* 18	S	I	R	I	I	S	R	I	I	S	I	**0.1**
*E. coli* 19	S	R	I	R	I	R	R	R	S	R	R	**0.6**
*E. coli* 20	S	I	S	S	S	S	I	S	S	S	I	**0**
*E. coli* 21	S	R	R	S	I	R	I	I	S	R	S	**0.3**
*E. coli* 22	S	R	R	I	R	R	R	R	I	R	I	**0.5**
*E. coli* 23	R	R	S	I	I	R	S	R	R	S	R	**0.5**
*E. coli* 24	S	S	R	S	S	S	R	S	S	S	I	**0.1**
*E. coli* 25	S	R	R	R	S	R	R	S	S	R	R	**0.6**
*Total R%*	**8%**	**60%**	**44%**	**36%**	**12%**	**48%**	**56%**	**20%**	**12%**	**48%**	**40%**	

### Morphology of phage vB_Ec_ZCEC14

**Fig. 1:**
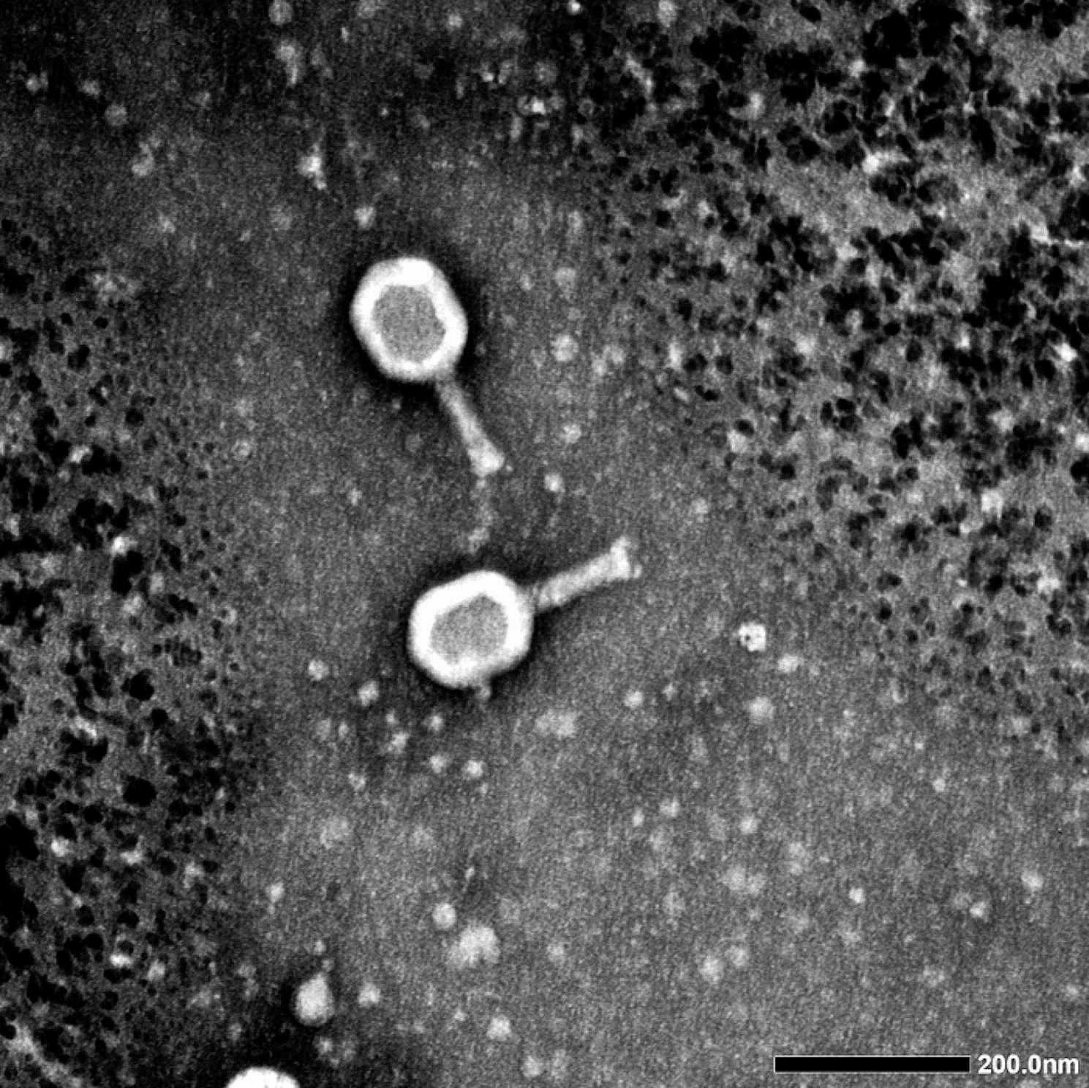
Morphology of phage vB_Ec_ZCEC14 by Transmission electron microscope

The TEM images of phage vB_Ec_ZCEC14 indicated that phage vB_Ec_ZCEC14 exhibits an icosahedral head (∼130 nm diameter) and a contractile tail (∼120 nm) with a base plate, as depicted in Fig. 1.

### Host range and efficiency of plating (EOP)

Phage host range was investigated against 25 *E. coli* isolates recovered from urine clinical samples and 10 *E. coli* isolates from the ECOR collection. It was able to produce lysis zones (≥ 20 plaques) against 11 out of 25 *E. coli* isolates and to *E. coli* O25 out of the 10 ECOR strains, which reflects broad range infectivity. Various strains of *E. coli* were found to exhibit a range of EOP when exposed to phage vB_Ec_ZCEC14. Among the eleven isolates of *E. coli* that were susceptible, it was observed that three of them exhibited an EOP value ≥ 1, while the remaining eight isolates demonstrated an EOP value < 1.

### Frequency of BIMs

The BIMs were measured to determine the long-term lysis efficiency of phage vB_Ec_ZCEC14. The BIMs were retrieved subsequent to a high multiplicity infection of 100 in the host *E. coli* strain *EC*/4 with phage vB_Ec_ZCEC14 at 37 °C. The BIM frequencies were determined to be 0.0043 ± 0.0022.

### In vitro investigation of phage vB_Ec_ZCEC14

**Fig. 2:**
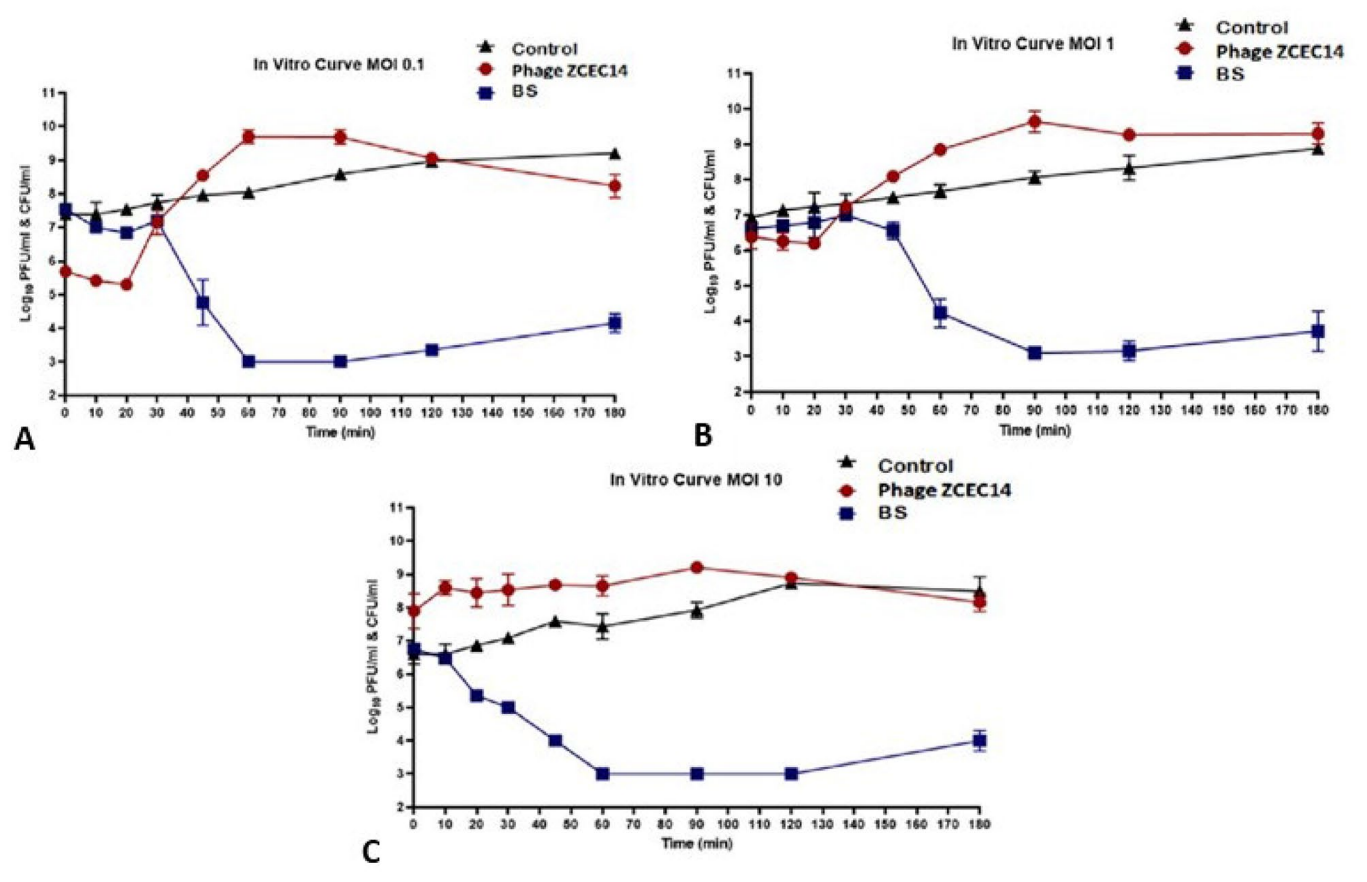
These figures illustrate the in vitro data of vB_Ec_ZCEC14 at 37 °C. They represent the bacterial and phage titers of *EC*/4 infected with vB_Ec_ZCEC14 at (**A**) MOI 0.1, (**B**) MOI 1, and (**C**) MOI 10. MOI: Multiplicity of infection; BS: Bacterial Survival

The dynamics and characteristics of the bacteriolytic activity of phage vB_Ec_ZCEC14’s was investigated against the host bacteria *EC*/4 at different MOIs (0.1, 1, and 10) over a period of 3 h (Fig. 2A and C). The bacteriolytic efficacy of phage vB_Ec_ZCEC14 was assessed through a comparative analysis of the growth reduction of the host EC/4 treated with phage, compared to the bacterial control without phage. The viable bacterial count was initially 6.7 log_10_ CFU/mL at MOI 10 and 7.5 log_10_ CFU/mL at MOI 0.1. Phage vB_Ec_ZCEC14 took 60 min to make a significant decrease in viable bacterial count to ∼3 log_10_ CFU/mL at both MOI 10 (*P* < 0.0002) and MOI 0.1 (*P* < 0.0007), as shown in Fig. 2A, C. While it took 90 min to reduce the viable bacterial count from 6.7 log_10_ CFU/mL to ∼3 log_10_ CFU/mL at MOI 1 (*P* < 0.0006), as shown in Fig. 2B. A slight recovery of bacterial populations was observed after 120 min; however, it did not reach a one-log increase in the viable bacterial count at the end. A decrease in viable bacterial count was accompanied by an observable increase in phage titers at MOI 1 to 9 log_10_ and MOI 0.1 to 8 log_10_ (Fig. 2A, B), while it did not encounter significant measurable change at MOI 10 (Fig. 2C). At lower MOIs, phage replication was observed, coinciding with the initial decline in viable bacterial count. Based on the above results, phage vB_Ec_ZCEC14 showed potent bacteriolytic activity at different MOIs.

### Phage vB_Ec_ZCEC14 temperature, UV, and pH stability

**Fig. 3:**
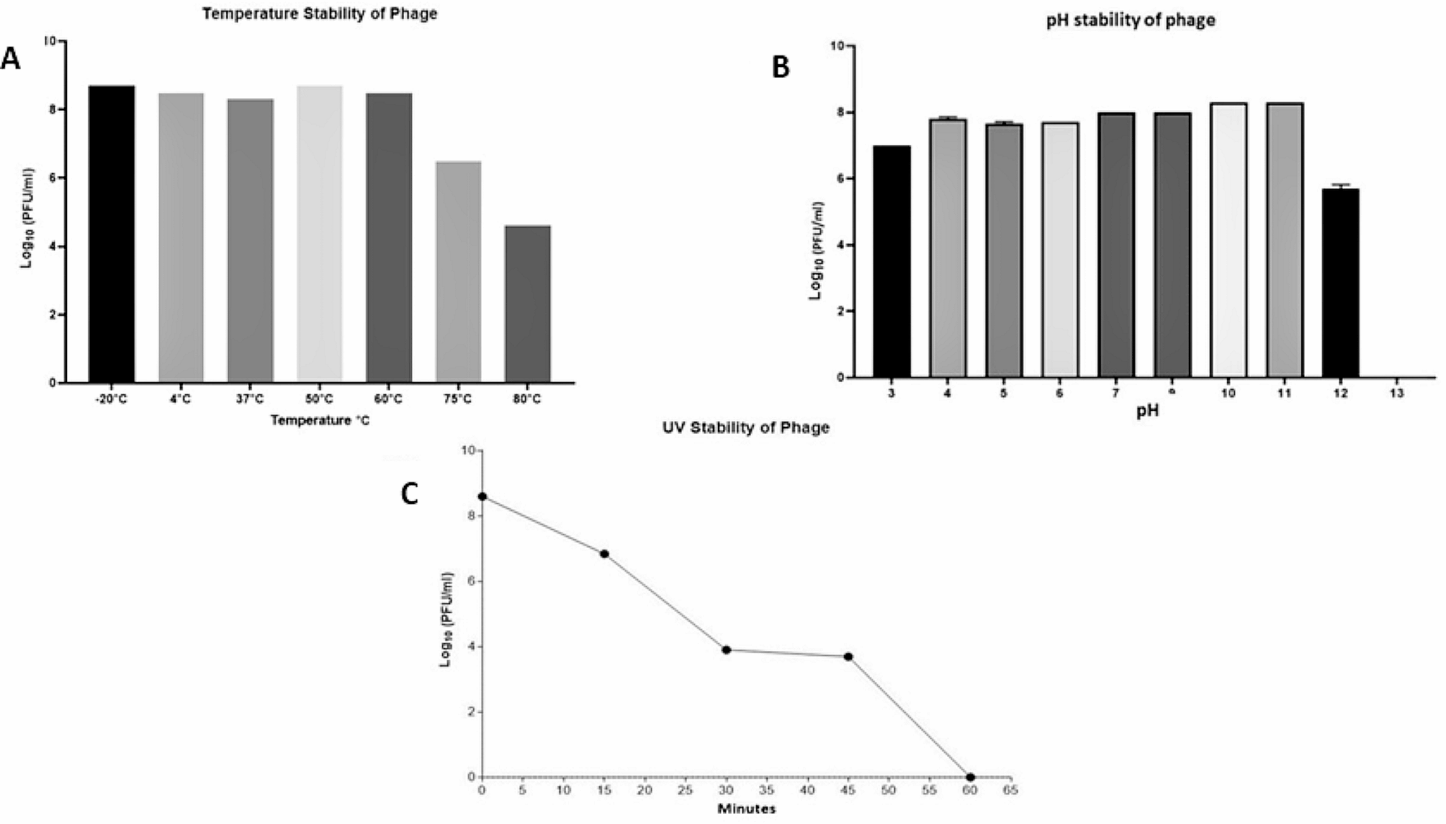
Phage vB_Ec_ZCEC14 viability at different ranges of temperature (**A**), pH (**B**), and UV exposure (**C**). PFU: Plaque forming unit

Evaluation of phage endurance at broad range of temperatures, exposure periods to UV, and pH is an essential assessment for its applicability as a biocontrol agent. Accordingly, the stability of vB_Ec_ZCEC14 was evaluated at various temperatures, pH values and UV conditions. Compared to the initial phage titer (10^8^ PFU/ml), phage vB_Ec_ZCEC14 was stable with its initial titer (10^8^ PFU/ml) for 60 min at the following temperatures − 20, 4, 37, 50, and 60 °C. Higher temperatures 75 ℃ and 80 ℃significantly dropped phage titers to 10^6^ PFU/mL and 10^4^ PFU/ml, respectively, which proves the endurance of phage vB_Ec_ZCEC14 to high temperatures, as shown in Fig. 3A. The stability of phage vB_Ec_ZCEC14 was evaluated by subjecting it to varying pH levels. It exhibits optimal stability over a pH range of 7.0 to 11.0, thereby retaining its initial titer of 10^8^ PFU/ml. At acidic pH values (pH 3.0 to pH 6.0), phage titers encountered a one-log reduction, while at pH 12.0, phage titer significantly dropped to 10^5^ PFU/ml. Phage vB_Ec_ZCEC14 was observed to be rendered entirely inactive upon being subjected to pH 3.0 and pH 13.0, and titers declined below detection limit, as shown in Fig. 3B. The stability of phage vB_Ec_ZCEC14 (10^8^ PFU/ml) was evaluated over different time periods of UV exposure 15, 30, 45, and 60 min. Phage titer decreased after 15 min of exposure to UV to 10^6^ PFU/ml. The results depicted in Fig. 3C indicated that phage titer is reduced to 10^4^ PFU/mL after 30 min and even for 45 min. The phage was inactive after 60 min of exposure to UV and its titer was below the limit of detection, as illustrated in Fig. 3C.

### DNA sequencing, assembly, and annotation for phage vB_Ec_ZCEC14

**Fig. 4:**
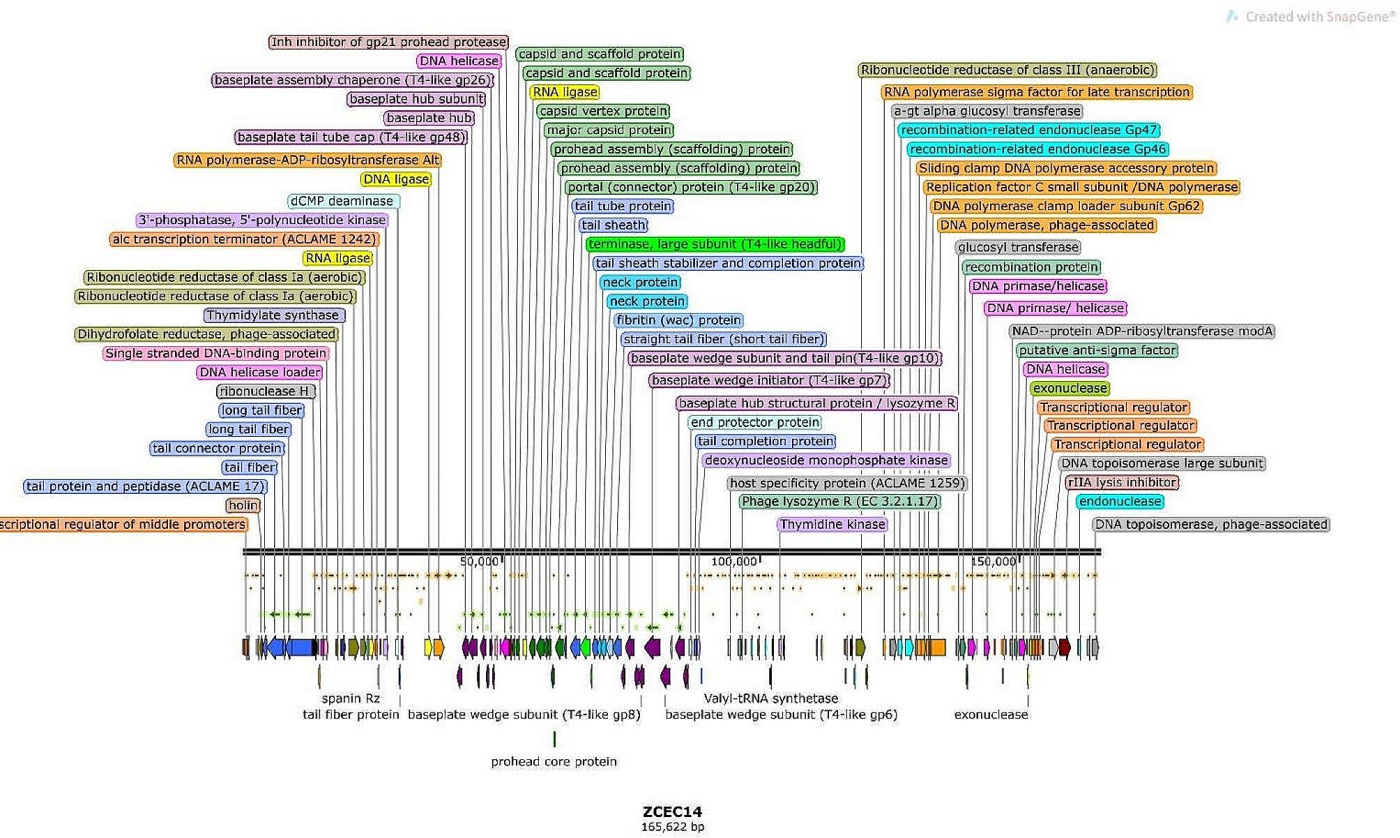
Genetic map of vB_Ec_ZCEC14 highlighting coding sequences with assigned function. Some functional genes were not illustrated due to space limits

**Fig. 5:**
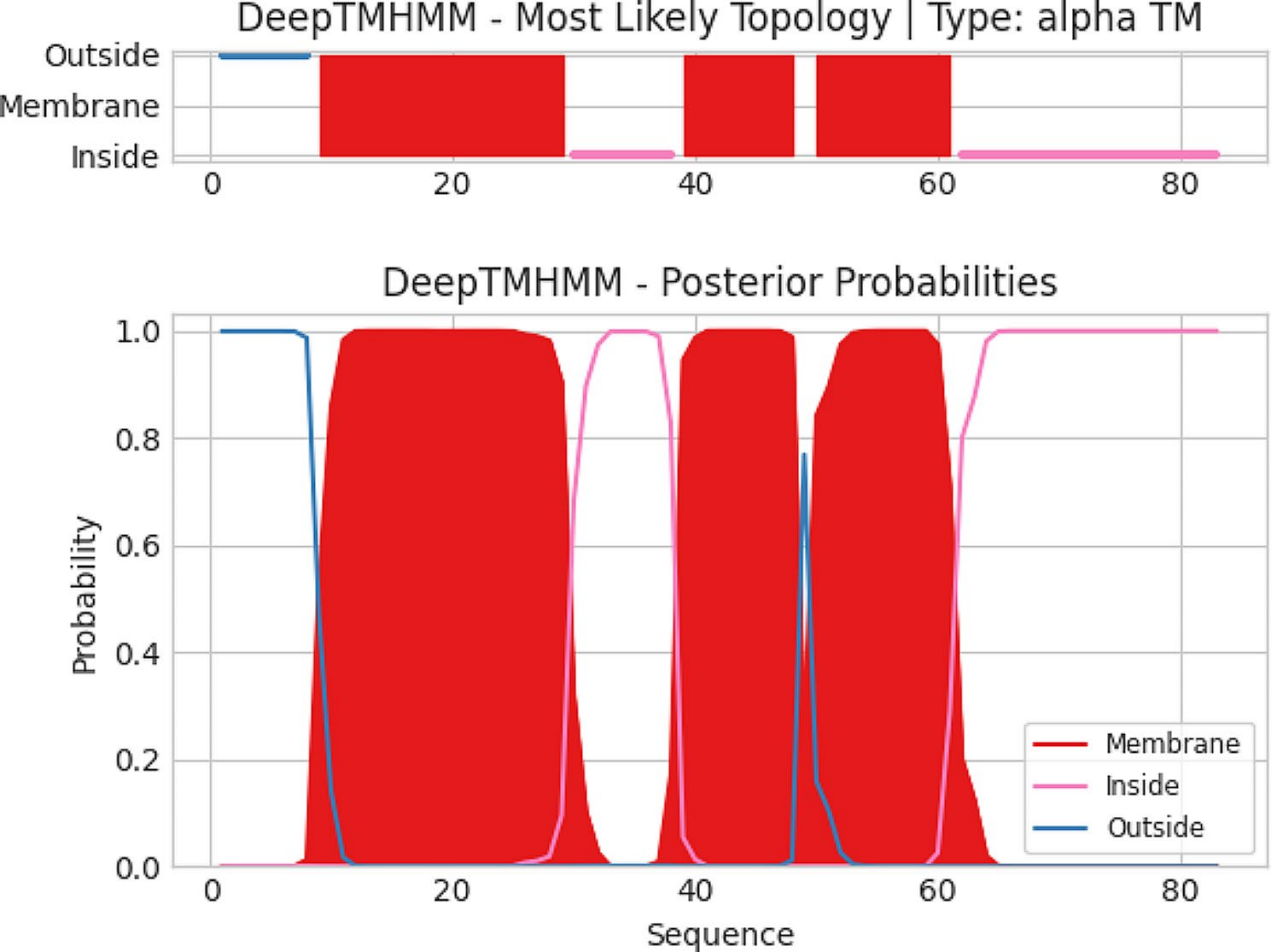
Transmembrane topology in immunity protein (ORF 212). X-axis: sequence position of amino acids; Y-axis: prediction probability; Red blocks: transmembrane domains; Blue line: domains outside the membrane; Pink line: domains inside the membrane

In consistence with the PFGE results, DNA sequencing of vB_Ec_ZCEC14 genome revealed a double-stranded DNA of ∼166 kbp length. Then, the phage genome was submitted into GenBank (Acc. No. OP168898). Genome assembly showed the following metrics: contigs no. 1; GC% 48.87; N50 165,622; L50 1; and 0 N’s per 100 kb. Two hundred and sixty-five protein-coding genes were predicted through integration of different annotation platforms, among which one hundred and forty-seven proteins have allocated functions linked to DNA replication transcription/packaging/repair, cell lysis, and structural phage proteins (Supplementary Table S1). Furthermore, phage vB_Ec_ZCEC14 did not encode any lysogenic genes such as integrase or transposases. On the plus strand, phage vB_Ec_ZCEC14 has two hundred and twenty-three ORFs, while it has only forty-two ORFs on the complementary strand. Further genetic details are illustrated on the genetic map which highlight functional genes as shown in Fig. 4. Genomic analysis uncovered two repeat regions and ten tRNA (Arg, Asn, Gln, Gly, Leu, Met, Pro, Ser, Thr, and Tyr). PhageLeads screened the genome without detection of any genes with the potential of antibiotic resistance, lysogenic lifecycle, or bacterial virulence. This indicates the applicability of vB_Ec_ZCEC14 in therapeutic purposes. Regarding transmembrane domains, they were detected in thirteen proteins using DeepTMHMM. Three TMDs was only predicted in immunity protein (ORF 212) (Fig. 5). While two TMDs were recorded in seven proteins (ORFs: 117, 126, 161, 168, 184, 213, and 226), and one TMD was recorded in five proteins (ORFs: 11, 65,103, 259, and 260).

### Phylogenetic analysis of phage vB_Ec_ZCEC14

**Fig. 6:**
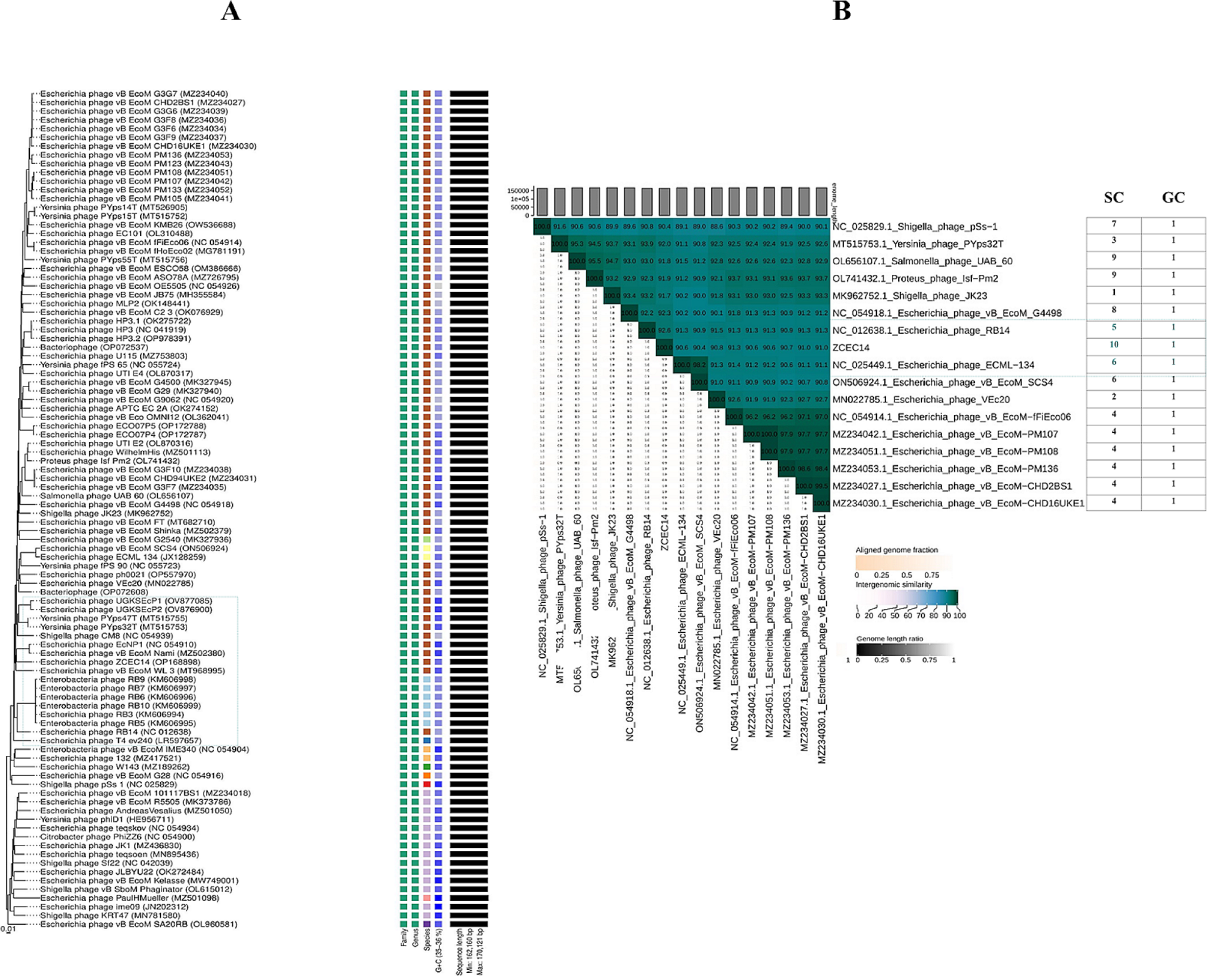
Phylogenetic analysis of vB_Ec_ZCEC14. (**A**) VICTOR phylogenetic tree of vB_Ec_ZCEC14 and top 100 BLASTn closely related phages; (**B**) VIRIDIC heatmap of phage vB_Ec_ZCEC14 and sixteen BLASTn closely related phages

**Fig. 7:**
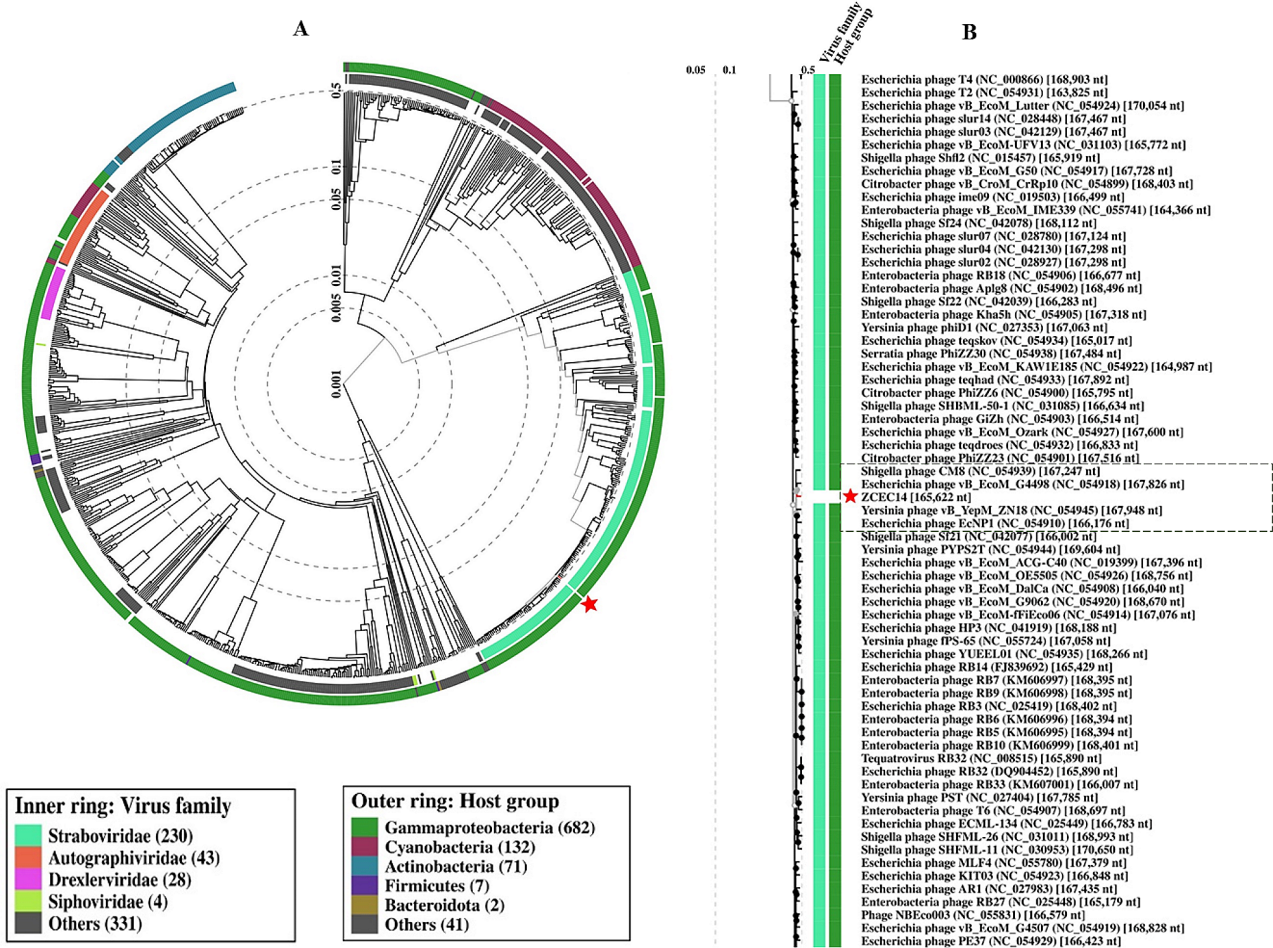
ViPTree proteomic tree of vB_Ec_ZCEC14 against RefSeq genomes of closely related phages. (**A**) Circular tree; (**B**) Rectangular tree illustrates a portion in the circular tree showing closely related phages

**Fig. 8:**
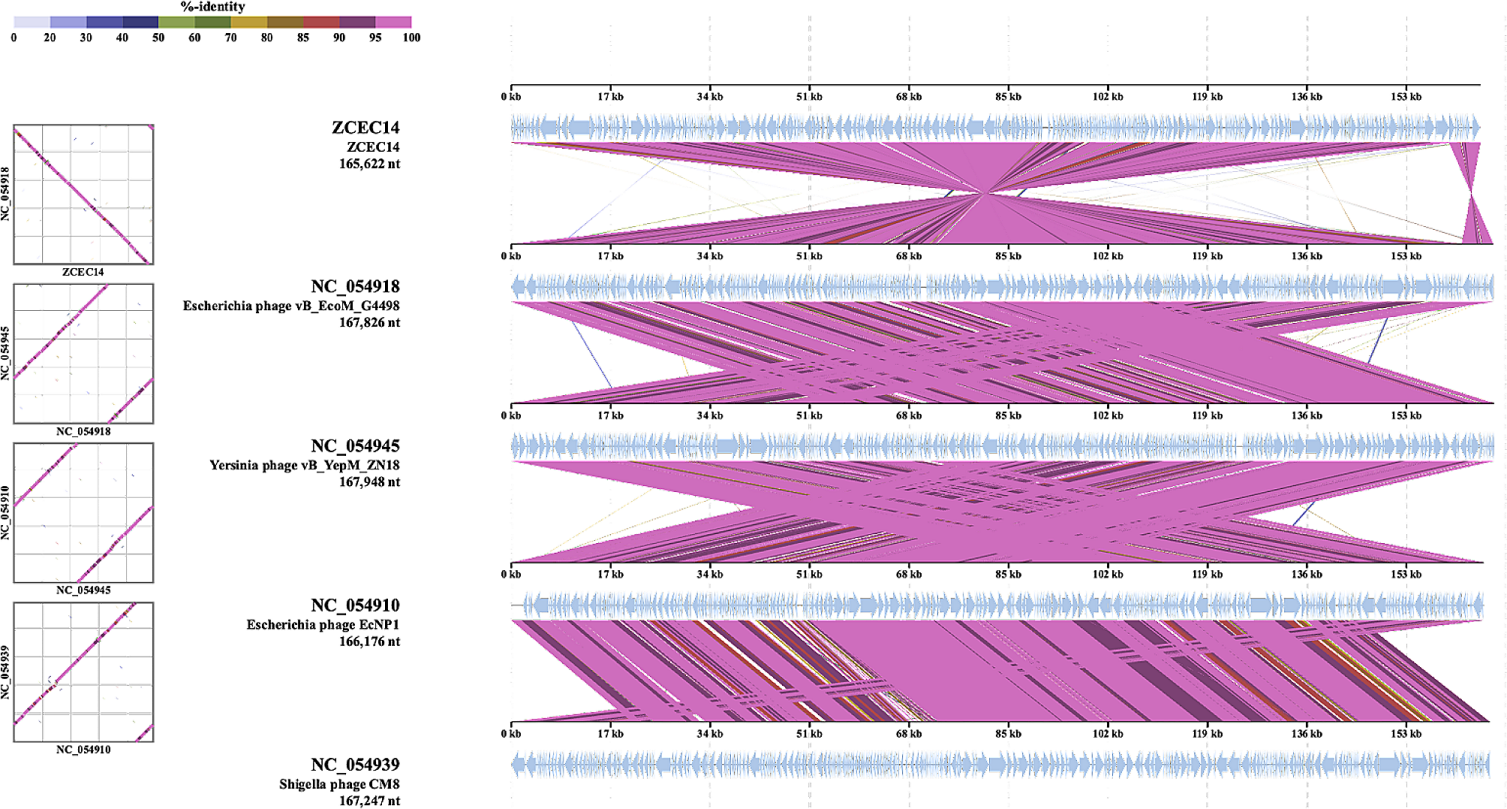
Complete genome comparison of vB_Ec_ZCEC14 and ViPTree closely related phages

**Fig. 9:**
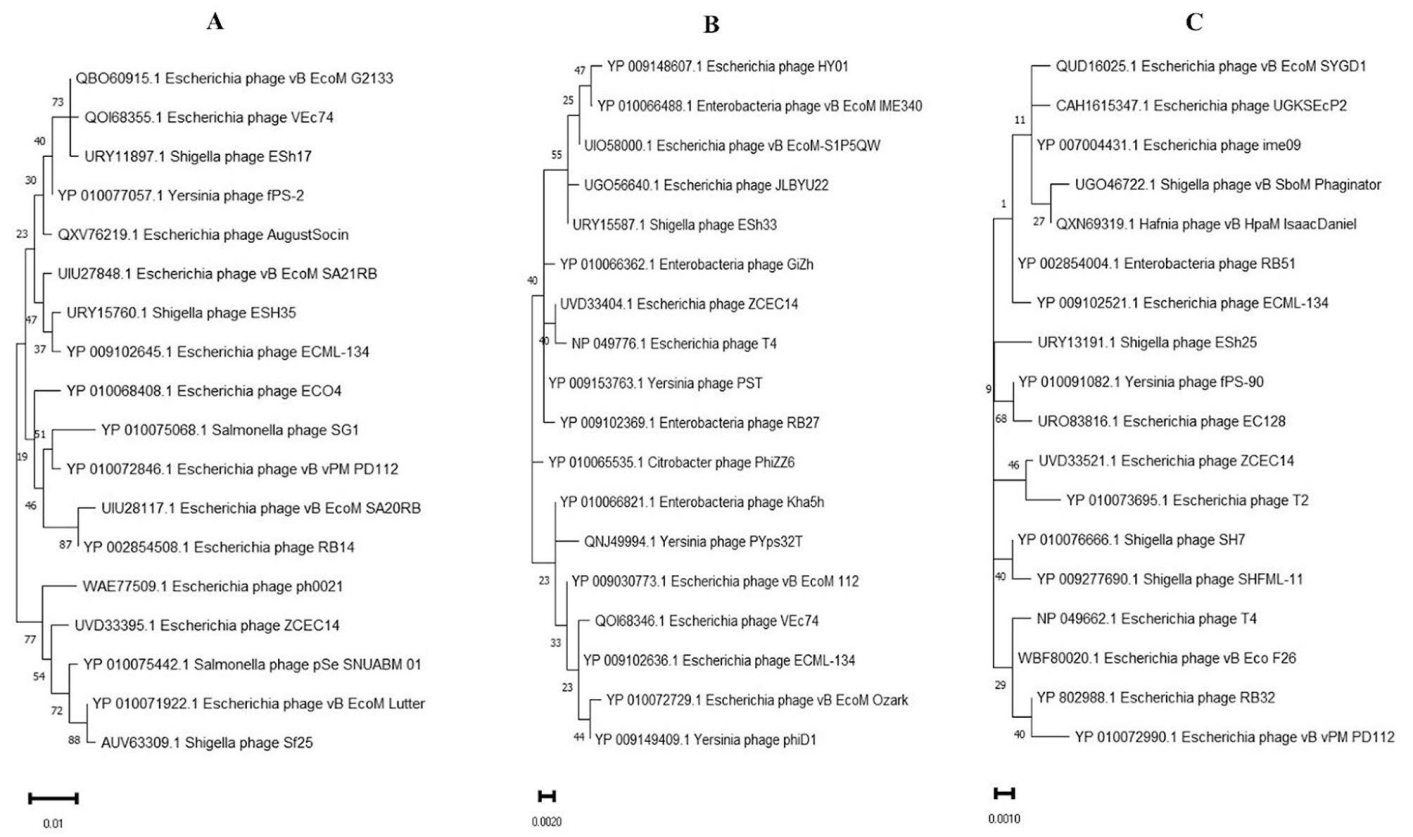
Phylogenetic analysis of the three signature proteins of vB_Ec_ZCEC14 and top BLASTp matched of other phages using MEGA-11. (**A**) major capsid protein; (**B**) terminase large subunit (TerL); (**C**) DNA polymerase

The phylogenetic analysis conducted by VICTOR following phylogenomic GBDP platform has been inferred from D6 formula yielding 0% average support (Fig. 6A). OPTSIL clustering of VICTOR analysis yielded twelve species clusters, and one cluster over the generic and family levels. Phage vB_Ec_ZCEC14 clustered with closely related *Escherichia* phages vB_ECOM_Nami, vB_ECOM_WL-3, and EcNP1. VIRDIC calculated intergenomic similarities between vB_Ec_ZCEC14 and top BLASTn matched phages. *Escherichia* phages RB14 and RCML 134 were clustered in the same species and genus thresholds with vB_Ec_ZCEC14 among the shortlisted 16 phages (Fig. 6B). Additionally, the proteomic tree generated by ViPTree confirmed that vB_Ec_ZCEC14 is a member of the *Straboviridae* family, in the class *Caudoviricetes* and grouped with other *Escherichia* phages (Fig. 7A, B). As well, ViPTree inferred that vB_Ec_ZCEC14 is highly similar to *Escherichia* phages vB_ECOM_G4498 and EcNP1, *Shigella* phage CM8, and *Yersinia* phage vB_YepM_ZN18. The complete genome of these closely related phages was aligned and compared to vB_Ec_ZCEC14 highlighting the similarities and differences (Fig. 8). Coregenes analysis was performed using some selected closely related *Escherichia* phages (RB14, ECML-134, vB_EcoM_G4498, and vB_EcoM_SCS4). Based on pan-genome analysis, vB_Ec_ZCEC14 shared two hundred and thirty-five orthologous genes. Three signature genes (major capsid protein, large terminase subunit (Terl), and DNA polymerase) were chosen to perform sequence alignment, and further phylogenetic analysis in MEGA-11 (Fig. 9A-C). In the three trees, signature genes of phage vB_Ec_ZCEC14 were clustered with those of other *Escherichia* phages. Following recent updates of International Committee on Taxonomy of Viruses 2023 (ICTV), genomic characteristics and host type of phage vB_Ec_ZCEC14 fit the family *Straboviridae* of the class *Caudoviricetes*.

## Discussion

Antibiotic resistance has become a global threat in the last years and the infections caused by antibiotic-resistant bacteria such as UTIs are becoming a great challenge to the health care sector. This raised interest in alternatives to antibiotics including bacteriophages. Our study aimed at isolating a new phage to tackle UTIs caused by *E. coli*. Phage vB_Ec_ZCEC14 effectively lysed twelve isolates out of 35 confirmed *E. coli* isolates. According to the morphological analysis and sequencing data of phage vB_Ec_ZCEC14, it belongs to *Straboviridae* family, with a head capsid, a contractile tail, and a 166 kbp genome size. This description is compatible with the previously reported studies about *Straboviridae E. coli* phage T4, that was reported to have 168 kbp approximate genome size and similar morphological features [73, 74]. Phage vB_Ec_ZCEC14 has maintained its thermal stability from the least temperature tested which is -20 ℃ to 60 ℃, and its titer decreased by two logs when incubated at 70 ℃. After incubation at 80 °C, phage vB_Ec_ZCEC14 titer was $$ {10}^{4}$$ PFU/ml. *E. coli* phages investigated in other studies, such as ZCEC10, ZCEC11, and ZCEC12, exhibited remarkable thermal stability from − 20 ℃ to 75 ℃, however, they were rendered completely inactive at 80 ℃ [75]. The optimum pH range for phage vB_Ec_ZCEC14 activity was between 7.0 and 11.0. Phage vB_Ec_ZCEC14 was inactive when incubated at pH 2.0 and pH 13.0. Moreover, phage vB_Ec_ZCEC14 showed high stability against UV exposure for 45 min. The replication dynamics studies of phage vB_Ec_ZCEC14 have demonstrated its ability to control the growth of *EC*/4 and reduce its viable bacterial count. The replication dynamics of phage vB_Ec_ZCEC14 demonstrated that its effectiveness in controlling the growth of *EC*/4 is concentration independent. Unlike phages ZCEC10, ZCEC11, and ZCEC12, which reduced the proliferation of *E. coli* O18 in a concentration-dependent manner at 0.001 to 10 MOI range, phage vB_Ec_ZCEC14 significantly reduced the bacterial population by ∼3 log_10_ CFU/mL at MOIs 10 and 0.1 after 60 min. A similar reduction was observed at MOI 1, but after 90 min. Phage titers increased by 4 log_10_ PFU/ml at MOI 0.1 and by 3 log_10_ PFU/ml at MOI 1, while a slight increase was observed at MOI 10, which may be due to the lysis from without (LO_V_). Lysis from without is identified as the early lysis of bacteria induced by phage adsorption of high multiplicity without producing new phages [76]. Consequently, the bacterial count was promptly reduced without apparent increase in the phage progeny at MOI 10.

In our study, six pathogenicity genes of *E. coli* (traT, fimH, blaCTX, blaSHV, blaTEM, and tetA) were tested against a selection of eight multidrug-resistant urine isolates that were sensitive to phage vB_Ec_ZCEC14 [77]. Many studies have revealed a high incidence and strong correlation between *fimH* and uropathogenic isolates [78, 79]. According to Abd Elbaky et al., *fimH* was recorded in 75% of tested *E. coli* isolates, with 77.7% prevalence rate [80]. The *fimH* gene is required in the bacterial infection process, where it is expressed as d-mannose-specific adhesion, type 1 fimbriae. Uropathogenic *E. coli* has more *fim*H, hlyA, *csg*A, *crl*, and traT genes than ExPEC and intestinal isolates because uropathogenic *E. coli* are more virulent than other studied isolated strains [81]. The *traT* virulence gene encodes the *traT* protein, which is a key factor in UTIs development through inducing serum resistance mechanism [82]. Serum-resistant *E. coli* are virulent as they may evade the immune complement system resulting in boosting of serum survival raising the prevalence of septic shock and death [83]. In this research, we reported *traT* gene in 77.7% of the multi-drug-resistant urine isolates. This matches the data obtained by Firozeeh et al. where *traT* gene was the most abundant virulence gene among all UPEC isolates collected from cystitis (67.9%) and pyelonephritis (80.6%). The *traT* gene is also common among urine *E. coli* isolates (59.1%) [84, 85]. Resistance of pathogenic *E. coli* to beta-lactam is often encountered by the degradative enzyme beta-lactamase, and the therapy options for extended spectrum beta-lactamase (ESBL) producing pathogens are restricted globally [86, 87]. *SHV, CTX-M*, and *TEM* are the most common ESBL genes, which mediate resistance to different classes of beta-lactam antibiotics and exist in a broad spectrum of clinically significant bacterial infections [87]. Before the 2000s, SHV and TEM were the most prevalent ESBL types; however, *CTX-M* enzymes have turned to be more prevalent in recent decades [88]. The elevated levels of *CTX-M* enzymes in the bacterial isolates are thought to be attributable to a high level of gene mobilization [89]. Barlow et al. demonstrated that there was a ten-fold increase in transfer of *blaCTX-M* genes encoded in a plasmid relative to other A-lactamases class [88, 90]. A genetic investigation of 115 uropathogenic *E. coli* isolates, which were collected from hospital-acquired infections, revealed that the most common gene was *TEM* (78.2%), followed by *blaCTX*-M (66%) and *blaSHV* (43.4%) [91]. This coincides with our findings, as *blaCTX* was the most found gene among the four tested resistance genes. The frequencies of *blaCTX*, *blaTEM*, and *blaSHV* in a selection of MDR *E. coli* isolates, which were sensitive to phage vB_Ec_ZCEC14, were 66.6%, 55.5%, and 55.5% respectively. An active efflux, mediated by an export protein is considered the cause of resistance to tetracyclines in Gram-negative bacteria [92]. Several tetracycline efflux genes have been characterized in Gram-negative bacteria, and the classification of strongly related genes was based on hybridization testing or gene sequencing [93]. In this study, *tetA* was screened in a selection of urine *E. coli* isolates, that were sensitive to phage vB_Ec_ZCEC14 and was detected at a rate of 11.11%. Our findings will provide extra benefits for competent authorities and clinicians to use phages in the fight against bacterial infections in the urinary tract. In addition, these results will boost the acknowledgement of bacteriophages in western societies and expedite the regulatory process of phage therapy.

### Electronic supplementary material

Below is the link to the electronic supplementary material.

**Supplementary Material 1: Table S1.** Phage vB_Ec_ZCEC14 genome annotation

## Data Availability

The dataset presented in this study can be found in online repositories. The genome sequences were deposited in GenBank under the accession numbers OP205141 and OP168898.
